# Metformin-like Methylglyoxal Scavengers from Macroalgae *Chondrus crispus* and *Gracilaria vermiculophyla* Preserve Cell Viability

**DOI:** 10.3390/md24050169

**Published:** 2026-05-09

**Authors:** George S. Hanna, Menny M. Benjamin, Latarsha Porcher, Sriram Vijayraghavan, Natalie Saini, Mark T. Hamann

**Affiliations:** 1 Department of Public Health Sciences, Medical University of South Carolina, Charleston, SC 29425, USA; 2 Department of Biomedical Sciences and Drug Discovery, Medical University of South Carolina, Charleston, SC 29425, USA; 3 Hollings Cancer Center, Medical University of South Carolina, Charleston, SC 29425, USA

**Keywords:** qNMR, methylglyoxal, metformin, natural products, macroalgae, guanylurea, AGEs

## Abstract

A quantitative NMR assay was utilized to identify methylglyoxal-scavenging natural products from Rhodophytes, *Chondrus crispus* and *Gracilaria vermiculophylla*. This revealed the activity of guanylurea-containing amino acid derivatives, gongrine and gigartinine. The molecules share structural features with the frontline blood glucose-lowering drug and plant natural product derivative, metformin, and scavenge methylglyoxal via the same mechanism, resulting in an imidazole-containing Advanced Glycation Endproduct or AGE. The protective effect of the molecules reported here was evaluated in a cell-based model for reactive aldehyde stress using methylglyoxal exposure to reduce cell viability. Gongrine, gigartinine, and metformin all preserve cell viability in HepG2 following methylglyoxal exposure. This is the first report of methylglyoxal scavenging and cell viability protection of these macroalgae-derived guanylurea-containing natural products, which can be found in high abundance in commonly consumed and industrially produced macroalgae species. The compounds presented here, along with their algal sources, offer a unique opportunity to produce guanylureas with therapeutic potential through sustainable production methods from easily cultivated algal sources.

## 1. Introduction

Previous efforts using quantitative NMR (qNMR) to screen marine macroalgae for scavengers of the reactive aldehyde species (R-CHO) methylglyoxal (MGO) revealed positive activity for extracts from the rhodophyte species *Chondrus crispus* Stackhouse 1797, and two *Gracilaria* species [1]. In continuation of that work, an NMR-guided approach to aldehyde scavenging was used to identify active constituents from *C. crispus* and *Gracilaria vermiculophylla* (Ohmi) Papenfuss 1967, revealing the MGO scavenging activity of guanylurea-containing natural products that possess structural features similar to those of metformin. Metformin, which was conceptually derived from the natural product galegine, is among the most commonly prescribed pharmaceuticals worldwide, with prescriptions increasing substantially in recent years due to the identification of additional health benefits [2]. Its primary indication is for lowering of blood sugar in diabetics and prediabetics, but it is now being investigated as a cancer-suppressing agent, to treat polycystic ovary syndrome, and to slow the progression of neurodegenerative diseases, including Alzheimer’s and Parkinson’s disease [3]. The broad, pleiotropic effect of metformin may be due, in part, to its scavenging of R-CHO, including MGO, which would decrease systemic damage to proteins and DNA, thus inhibiting Advanced Glycation Endproduct (AGE) formation and slowing the progression of related diseases [4,5]. Despite its wide use in humans, the mechanism of action for metformin’s blood sugar-lowering effect is not fully understood. However, metformin has been shown to react with MGO in humans and lower its circulating concentration, which may contribute to its therapeutic effect [6]. Due to the widespread and growing use of metformin at relatively high dosages and by a large population (estimated 200 million globally), along with its persistence in the environment, there has been a growing set of concerns regarding its potential environmental impact [7,8].

**Figure 1:**
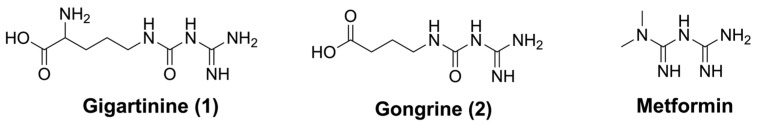
Structures of gigartinine, gongrine, and metformin.

First reported in “Nature” in 1966, novel guanylurea-containing amino acids, gigartinine (1) (L-α-ammo-δ-(guanylureido)-n-valeric acid) and gongrine (2) (γ-(guanylureido)butyric acid, were discovered from *Gymnogongrus flabelliformis*, collected in Japan (Figure 1) [9,10]. Guanylurea-containing amino acid derivatives have been reported in high abundance in several species. For example, they make up 48.3% of total organic nitrogen in *G. flabelliformis*, although with significant seasonal fluctuations [9,11]. This class of molecules is considered exclusive to Rhodophyta, which possess a greater average protein concentration percentage in dry weight compared to other macroalgae with free amino acids (FAAs) comprising up to 55% of total tissue nitrogen content [12]. The presence, absence, or difference in concentration of Rhodophyta-derived guanylureas has been utilized as a chemotaxonomic tool for identifying morphologically indistinguishable species, and their production has been speculated to have an ecological role in their invasiveness [9,10,13,14]. Gigartinine has been reported in the following species: *Chondrus crispus*, *Ahnfeltia plicata*, *Petrocelis middendorfii*, *Polyides rotundus*, *Vertebrata lanosa* formerly (*Polysiphonia lanosa*), *Rhodomela confervoides*, *Gymnogongrus fiabelliformis*, *Grateloupia livida*, *Gelidium amansii*, *Polyopes polyideoides*, *Polyopes prolifer* formerly (*Carpopeltis flabellata*), *Hypnea japonica*, and *Gracilaria textorii.* Until the data reported here, reporting the isolation of gongrine from *C. crispus*, gongrine had only been reported from *Grateloupia livida*, *Polyopes prolifer, Gracilaria textorii*, and *Gymnogongrus flabelliformis* [9,10,11,12,13,14,15,16,17,18,19].

**Figure 2:**
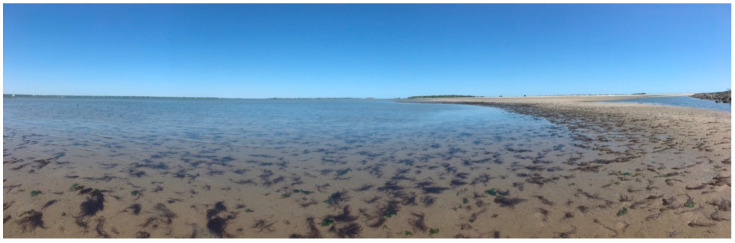
*G. vermiculophyla* shown in a tidal mud flat as a prolific and invasive species at Fort Johnson, Charleston, SC.

The selected species for this study, which yielded (1) (*G. vermiculophylla*) and (2) (*C. crispus*), both possess characteristics that make them viable candidates for industrial production of guanylureas. *C. crispus*, also referred to commonly as Dulse or Irish Sea Moss, is widely and historically consumed directly as a food, typically powdered and added as a thickener or stabilizer, and refined as an industrial source of carrageenan. *G. vermiculophylla*, along with other *Gracilaria* species, is widely grown in Asia, predominantly China and Indonesia, with an annual production of 5 million tons in 2020, a dramatic increase from 2 million tons in 2010, where it is predominantly used for agar production and as an aquaculture feed for abalone [20]. *G. vermiculophylla* became the subject of interest for this study because of its widespread invasion of temperate coastlines around the world, where it becomes a highly abundant species on intertidal mudflats (Figure 2) [21,22,23]. Identifying an economically viable utility for this invasive species may facilitate the development of an industry to harvest and process it within its non-native range.

Consequently, metformin has a problematic environmental footprint from two perspectives: its presence in aquatic ecosystems and the energy requirement of its synthesis. Due to metformin’s status as one of the most widely prescribed drugs, combined with its incomplete metabolism, metformin has become one of the most ubiquitous pharmaceutical contaminants in global waters [24,25,26]. Metformin is the reaction product of dimethylamine and 2-cyanoguanidine, resulting in a multistep manufacturing process with high energy demands due to the need for high temperatures and pressures involved in the reaction processes. This is compounded by the required starting material, ammonia, which is produced via the Haber-Bosch method, a method that emits more carbon dioxide than any other chemical production process [27]. Despite its long history of use, the continued growth in metformin prescriptions and its persistence in the environment and substantial carbon footprint justify the development of alternatives. Such alternatives, if produced through processes designed with green chemistry considerations, such as circular processes, renewable feedstocks, reduced toxicity, or reduced nutrient pollutants, or that yield additional commodities, such as industrial or food products, could provide sustainable solutions that are also cost-competitive [28,29]. The species selected for this study possesses the potential to satisfy those characteristics, along with a history of industrial-scale production, thus representing an ideal profile for further development of metformin replacements or other guanylurea-related chemical products.

## 2. Results

### MGO Scavenging Activity and Protection of Cell-Viability

**Figure 3:**
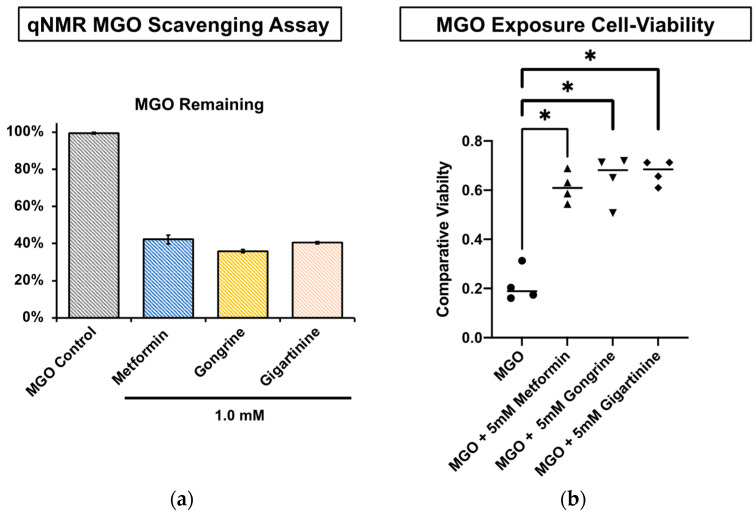
(**a**) MGO (1 mM) scavenging activity for 1 mM metformin, gongrine, and gigartinine incubated for 24 h at 37 °C in qNMR PBS buffer. (**b**) MGO exposure cell viability assay. * indicates *p*-value < 0.05 by a two-sided students *t*-test.

**Figure 4:**
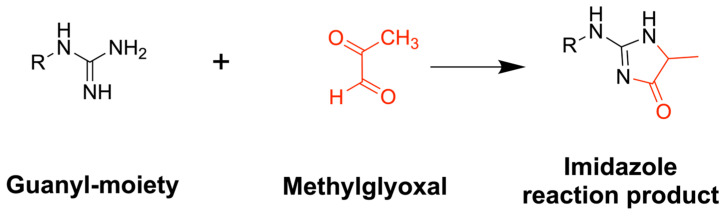
MGO scavenging reaction mechanism and structure of the major product.

Bioassay-guided isolation using a qNMR-based MGO scavenging method [1] resulted in the identification of gigartinine and gongrine as the major active constituents from *C. crispus* and *G. vermiculophyla* that reduce MGO concentrations. Following isolation, the MGO scavenging activity of gongrine and gigartinine was compared to that of the positive control, metformin. Metformin, gongrine, and gigartinine demonstrate comparable MGO scavenging activity, with 42.2%, 35.9%, and 40.5% MGO (1.0 mM) remaining after incubation at 37 °C for 24 h, respectively (Figure 3a). The HepG2 cell line was used, since these are nontumorigenic cells with epithelial characteristics and high proliferation capabilities. Treatment of HepG2 cells with 500 μM MGO for 24 h led to severe cytotoxicity. The CellTiter-Glo^®^ Assay (Promega, Madison, WI, USA) was used to determine cell viability. Data were normalized to untreated cultures marked at 100% viable (Figure S11). To determine the potential of NPs that scavenge MGO to preserve cell viability, HepG2 cells were treated with 5 mM MGO, or with 5 mM MGO and an equal amount of metformin, gongrine, or gigartinine, and cell viability was measured as described above. The protective effect of all three compounds was comparable, with a mean cell viability of 61.3% for metformin, 64.7% for gongrine, and 67.3% for gigartinine, compared to 21.3% for MGO alone (Figure 3b). Comparisons of the resulting NMR spectra show an analogous imidazole signal that forms when metformin, gongrine, and gigartinine react with MGO (Figure 4 and Figures S8–S10).

## 3. Discussion

It is important to highlight that guanylurea yields from macroalgae are subject to varied extraction and isolation methodologies, seasonal variability, and growing conditions, and total-yield calculations can be difficult due to solubility issues that complicate isolation chromatography. However, enrichment from crude extracts is achievable through precipitation [10,11,12,13]. Relevant to their production and ecology, dramatic seasonal fluctuations in concentrations were reported in Rhodophyta-derived guanylureas. For example, gigartinine composes 35% of the total nitrogen of *G. flabelliformis* harvested in December, but only 9% in January, and is entirely depleted during the summer months. Gongrine, on the other hand, reportedly fluctuates inversely with gigartinine, composing approximately 10% total nitrogen in the plant composition in December and 30% in January [9]. On average, gigartinine has a higher rate of prevalence and overall concentration in red algae in comparison to gongrine. Additional studies are required for a thorough understanding of their species distributions, temporal variability, and production in relation to environmental conditions [16]. There is evidence that suggests that the accumulation of guanylureas is correlated with the increase in nitrogen availability [12]. Nitrogen pulsing experiments were conducted to quantify variability in nitrogen metabolism and storage among different species. Following nitrogen dosing, it was found that *Gracilaria chilensis* was devoid of gigartinine, whereas morphologically similar *Gracilaria* sp. rapidly (within hours) accumulated gigartinine as a storage compound. In this case, gigartinine’s total FAA nitrogen concentration in *Gracilaria* was as high as 80% in culture [12,13]. An enhanced understanding of such variations, as well as utilizing proven methodologies to increase yields, such as strategic nitrogen pulsing, will make guanylurea-containing compounds excellent candidates to extract in high yield. Additionally, the reported high-yields of macroalgae guanylureas offer green chemistry opportunities using sustainable, carbon-negative algae aquaculture systems. There is unique potential to make an incremental change to carbon emissions by supplying one of the most frequently used drugs on the market through sustainable algal production while also mitigating anthropogenic nitrogen eutrophication.

These abundant molecules have been detected in several macroalgae species that are commonly eaten or processed into food products at industrial scales. It is feasible, based on the high yields and typical methods of food preparation of species, such as *Chondrus* sp., *Gymnogongrus* sp., or *Gracilaria* sp., that individuals could consume a dose of macroalgae-derived guanylureas equivalent to a typical low dose of metformin (0.5 g/day). This would only require approximately 10 g of dried macroalgae (~1–2 tablespoons) or approximately 100 g (1–2 cups) of fresh macroalgae. One such conceivable scenario would be the preparation of soup stock, which would align with traditional uses of the discussed species. Considering that hot water is an effective extraction method, historical human dietary exposure to gongrine and gigartinine is within reason and offers some insight into their safety. The unique attributes discussed above, combined with the MGO scavenging and cell-viability preserving activity presented here, make them interesting candidates as naturally produced metformin replacements or as dietary supplements for the control of the degenerative effects of circulating R-CHO, in particular, the formation of AGEs. With further development, potential therapeutic applications of the guanylureas include intercepting or slowing the progression of neurodegenerative diseases, cancer, cardiovascular disease, and other complications associated with hyperglycemia, which leads to elevated levels of MGO [5,30,31,32]. Further studies considering chronic MGO exposure and the administration of guanylureas in model systems for metabolic syndrome would make progress toward translation. The critical and widespread therapeutic application of metformin underscores its value, but its persistence in the environment and energy-intensive production methods are cause for consideration of approaches that apply green chemistry production principles. Presented here is evidence that guanylureas with similar structural and functional characteristics to metformin are produced by marine macroalgae, presenting an opportunity to derive replacements through more sustainable industrial practices.

## 4. Materials and Methods

Chemicals and Reagents. Deuterium oxide (D_2_O) was purchased from Cambridge Isotope Laboratories. HyClone, phosphate-buffered saline (PBS) (Cytiva, Marlborough, MA, USA, 0.0067 M PO_4_, pH 7.0–7.2), HPLC-grade methanol, and formic acid were purchased from VWR International (Radnor, PA, USA). Methylglyoxal (40%wt/vol in H_2_O), metformin, aminoguanidine, and dimethylsulfone (DMS—TraceCert qNMR Calibration Standard) were purchased from Sigma Aldrich (St. Louis, MO, USA). Ethanol (200 proof) was obtained through the Medical University of South Carolina and diluted to 70% with Milli-Q water.

Macroalgae Sourcing. *Chondrus crispus* was purchased from Gulf of Maine Inc. (Pembroke, MA, USA). *Gracilaria vermiculophylla* was collected at Fort Johnson during low tide and was characterized using Seaweeds of the Southeastern United States by Craig Schneider and Richard Searles. [33] Voucher specimens were obtained and stored at −80 °C.

Extraction, Sample Preparation, and Primary Screen. A previously established preparation protocol was used for the primary MGO scavenging screen [1]. Each sample was dried by lyophilization (Virtis Genesis, Gardiner, NY, USA, <2 Torr) and milled to a fine powder (CGoldenwall Grain Grinder, Ningbo, China). A total of 2.50 g of dried and milled material was added into a 50 mL centrifuge tube that was then filled with 70% Ethanol, shaken to ensure material was saturated with solvent, and sonicated for 2 h with periodic agitation of settled material. Each tube was centrifuged at 2000 rpm for 15 min and filtered through a Buchner funnel (Whatman Grade 1 filter paper, Maidstone, UK) into a 50 mL glass culture tube. All samples were then dried in vacuo (Thermo-Savant SpeedVac 210A concentrator, Waltham, MA, USA), transferred to vials, and weighed for stock solution preparation. This material was utilized for bioassay-guided isolation, with MGO scavenging serving as a positive indicator of activity.

Isolation of Active Molecules. The crude 70% EtOH extract of *G. vermiculophyla* and *C. crispus* that was used in the primary screening effort was suspended/dissolved in water and loaded onto a Sephadex C18 cartridge. A solid-phase extraction was conducted beginning with a desalting wash with 100% water and then with increasing increments of MeOH to generate 10%MeOH, 25%MeOH, 50%MeOH, and 100%MeOH fractions. The 10% EtOH fractions for *C. crispus* (CC-10) and the 10% and 25% EtOH fractions for *G. vermiculophylla* (GV-10, GV-25) exhibited the most MGO scavenging activity, with decreasing activity as EtOH concentrations increased (Figure S1). The qNMR data indicated distinct major constituents between the two species that warranted further isolation. Of note for the *G. vermiculophylla* results is the poor enrichment of the active constituent by C18 SPE cartridge—isolation work revealed the elution of these molecules even with 100% water during the desalting phase of the extraction, and poor resolution of the active molecule gigartinine under the employed conditions, which was addressed when scaled up for HPLC purification. This also prevented accurate total percent yields for the isolated molecules due to their presence in multiple fractions during the bioassay-guided isolation process. The active compound-containing fractions were then separated to isolate the active constituent using reversed-phase C18 chromatography with a gradient from 100% H_2_O to 30% Acetonitrile over 60 min with a flow rate of 20 mL/min and absorbance detection at 218 nm. Of importance is that gongrine demonstrates no functional absorbance at the typically used wavelength of 254 nm. Pure molecules, gongrine and gigartinine, were characterized by NMR (Bruker 600 MHZ) and LCMS/MS (Bruker Impact II) (Table S1, Figures S2–S7).

Gigartinine (1) (C_6_H_12_N_4_O_3_): ^1^H NMR (D_2_O, 600 MHz): **δ** 1.53 (*m*, 2H), 1.56 (*m,* 2H), 3.15 (*q*, 2H), 3.68 (*t*, 1H), 7.18 (*br,* NH). ^13^C NMR (D_2_O, 600 MHz): **δ** 24.5, 27.8, 39.1, 54.6, 154.5, 155.5, 174.6. m/z 218.14. NP-MRD ID: NP0351960.

Gongrine (2) (C_6_H_12_N_4_O_3_): ^1^H NMR (D_2_O, 600 MHz): **δ** 1.64 (*m*, 2H), 2.11 (*t,* 2H), 3.03 (*q*, 2H) ^13^C NMR (D_2_O, 600 MHz): **δ** 26.2, 34.9, 39.5, 160.9, 164.8, 183.0. m/z 189.11. NP-MRD ID: NP0351961 Poor solubility under neutral or acidic conditions impacted efforts for a quality ^13^C spectrum. To obtain a quality ^13^C spectrum, dilute ammonium hydroxide was added to improve solubility. There was a notable shift in the ^13^C signals for the guanylurea moiety at positions 5 and 6 from 155.5 ppm and 154.4 ppm in neutral or acidic conditions to the reported 160.9 ppm and 164.8 ppm in basic conditions.

Cell assays for methylglyoxal toxicity. HepG2 cells were grown in Eagle’s Minimum Essential Medium (EMEM) with 10% fetal bovine serum. Cells were seeded in 96-well plates at a concentration of approximately 500 cells per well. Cells were allowed to adhere and grow for 24 h. Methylglyoxal or a combination of methylglyoxal with the scavengers was added to the plates at the indicated concentrations. Four wells per treatment were used. Cells were incubated in the presence of the drugs for 24 h. The media was removed, cells were washed, and viability was measured using the CellTiter-Glo (Promega, Beijing, China) luminescent cell viability assay.

No GenAI was used in this manuscript.

## Data Availability

All NMR data have been uploaded to the Natural Products Magnetic Resonance Database.

## Supplementary Materials

The following supporting information can be downloaded at: https://www.mdpi.com/article/10.3390/md24050169/s1, Figure S1: MGO scavenging activity of *G. vermiculophylla* and *C. crispus* fractions generated by SPE using a C18 cartridge compared to the 1 mM MGO control and 0.5 mM metformin. Table S1: NMR Spectroscopic Data (600 MHz, D_2_O) for gigartinine and gongrine, Figure S2: ^1^H NMR spectra for gigartinine, Figure S3: ^13^C NMR spectra for gigartinine. Figure S4: LCMSMS EIC and fragment ion spectrum for gigartinine. Figure S5: ^1^H Spectra for gongrine, Figure S6: ^13^C Spectra for gongrine, Figure S7: LCMSMS EIC and fragment ion spectrum for gongrine, Figure S8: (A) Overlay of MGO control and 1 mM Metformin after incubation. (B) MGO signal used for quantification. (C) Indication of key signals (orange dots) and structure of Metformin-MGO reaction product based on previous reports, Figure S9: (A) Overlay of MGO control and 1 mM Gongrine after incubation. (B) MGO signal used for quantification. (C) Indication of key signals (orange dots) and putative structure of Gongrine-MGO reaction product, Figure S10: (A) Overlay of MGO control and 1 mM Gigartinine after incubation. (B) MGO signal used for quantification. (C) Indication of key signals (orange dots) and putative structure of Gigartinine-MGO reaction product, Figure S11: 5000 cells were seeded in a 96 well plate and treated with MGO (0, 100 μM to 5 mM) for 24 h. Cell viability was measured using CellTitre-Glo Assay. Each treatment was done in four replicates. X values were transformed using the function X = log(X) and a four-parameter non-linear fit was performed to calculate the IC50.

## Abbreviations

The following abbreviations are used in this manuscript:

qNMR	Quantitative Nuclear Magnetic Resonance Spectroscopy
R-CHO	Reactive Aldehyde Species
MGO	Methylglyoxal
AGE	Advanced Glycation Endproduct
FAA	Free Amino Acid
D_2_O	Deuterium oxide
PBS	Phosphate Buffered Saline
