# Supplementary material for: Metformin-like Methylglyoxal Scavengers from Macroalgae Chondrus crispus and Gracilaria vermiculophyla Preserve Cell Viability
Source: Mar Drugs. 2026 May 9;24(5):169. doi: 10.3390/md24050169 (PMC13208526; doi:10.3390/md24050169)
Supplement: Supplementary file 1 [file marinedrugs-24-00169-s001.zip › marinedrugs-4252909-supplementary.pdf]

Metformin-Like Methylglyoxal Scavengers from Macroalgae Chondrus crispus and Gracilaria vermiculophyla Preserve Cell Viability

George S. Hanna*[1], Menny M. Benjamin[2], Latarsha Porcher[3], Sriram Vijayraghavan[3], Natalie Saini[3], and Mark T. Hamann[2*]

[1]Medical University of South Carolina, Department of Public Health, Charleston, SC 29425.

[2]Medical University of South Carolina, Department of Biomedical Sciences and Drug Discovery, Charleston, SC 29425.

[3]Medical University of South Carolina, Hollings Cancer Center, Charleston, SC 29425

Corresponding author: *hannag@musc.edu;

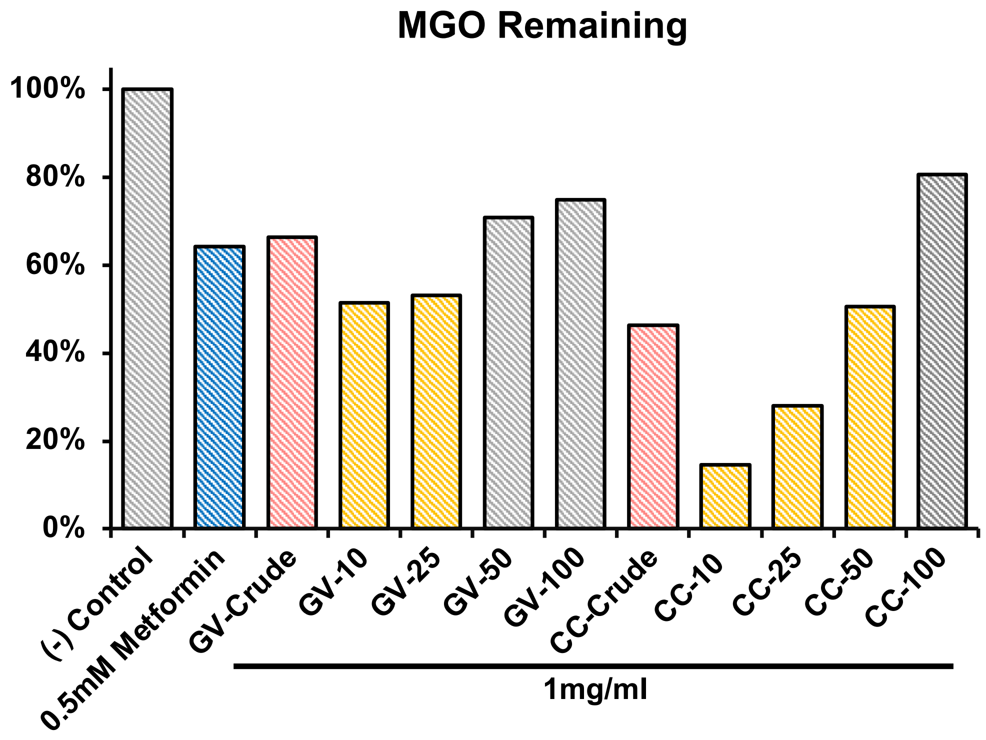

Figure S1: MGO scavenging activity of *G. vermiculophylla* and *C. crispus* fractions generated by SPE using a C18 cartridge compared to the 1mM MGO control and 0.5mM metformin.

Table S1: NMR Spectroscopic Data (600 MHz, D_2O) for Gigartinine and Gongrine

	Gigartinine **(1)**		Gongrine **(2)**	
position	δ_C, type	δ_H (J in Hz)	δ_C, type	δ_H (J in Hz)
1	174.6, C	-	183.0, C	-
2	54.6, CH	3.68, t (6.0)	34.9, CH_2	2.11, t (15.4)
3	27.8, CH_2	1.56, m (18.4)	26.2, CH_2	1.64, m (7.35)
4	24.5, CH_2	1.53, m (18.5)	39.5, CH_2	3.03, q (6.81)
5	39.1, CH_2	3.15, q (6.4)	160.9, C	-
6	155.5, C	-	164.8, C	-
7	154.4, C	-	n/a	-
NH (1)	-	-	-	-
NH (2)	-	-	-	-
NH (3)	-	7.18, brd	-	-
NH_2 (1)	-	-	-	-
NH_2 (2)	-	-	-	n/a

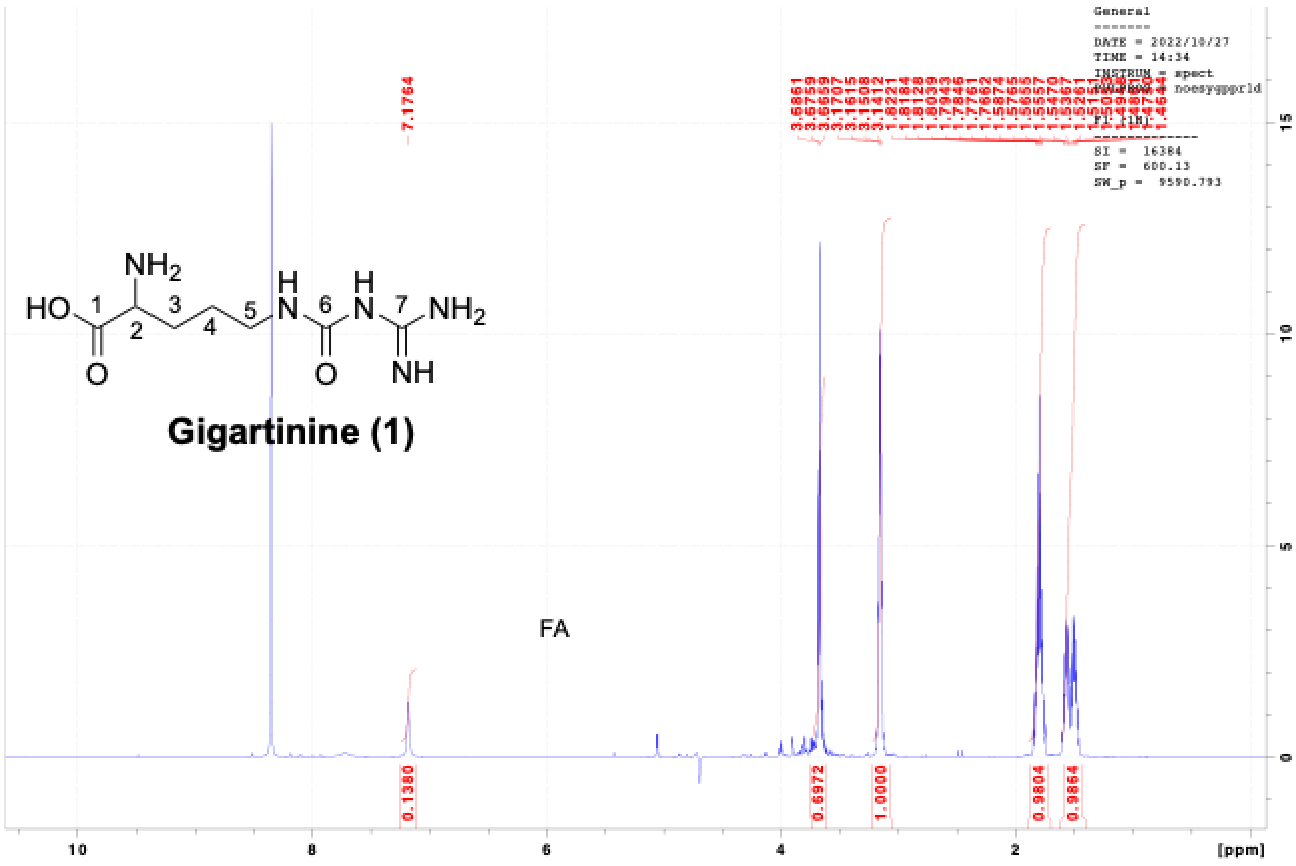

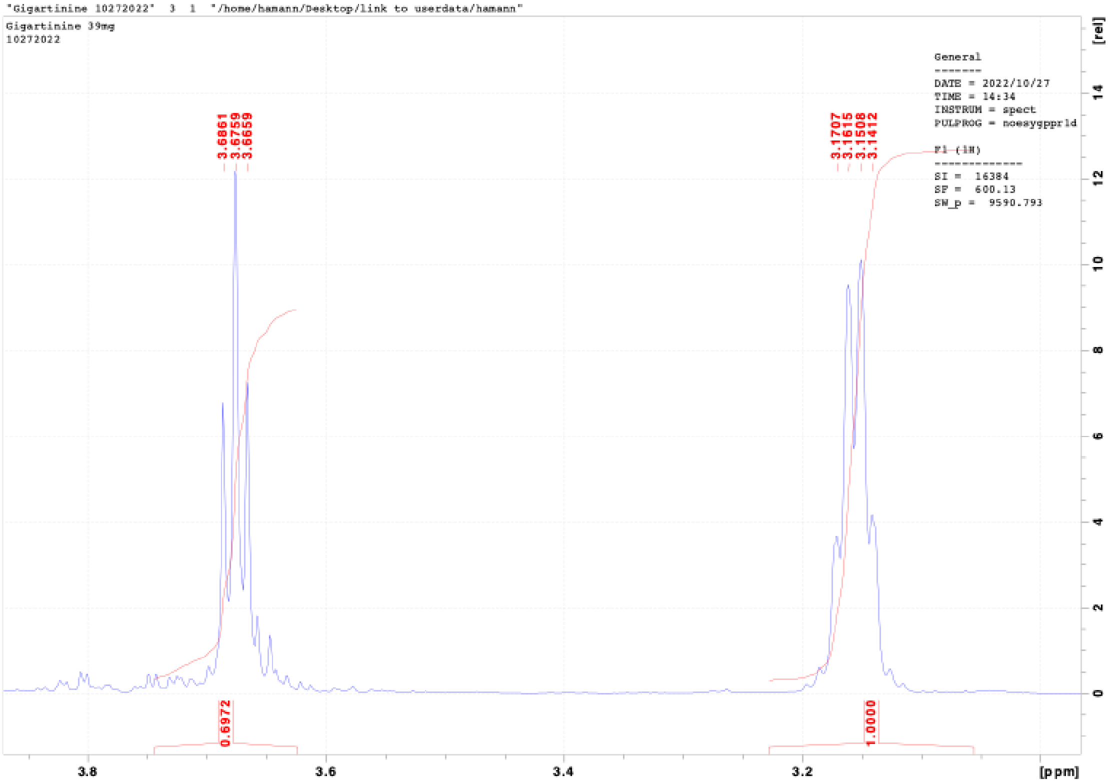

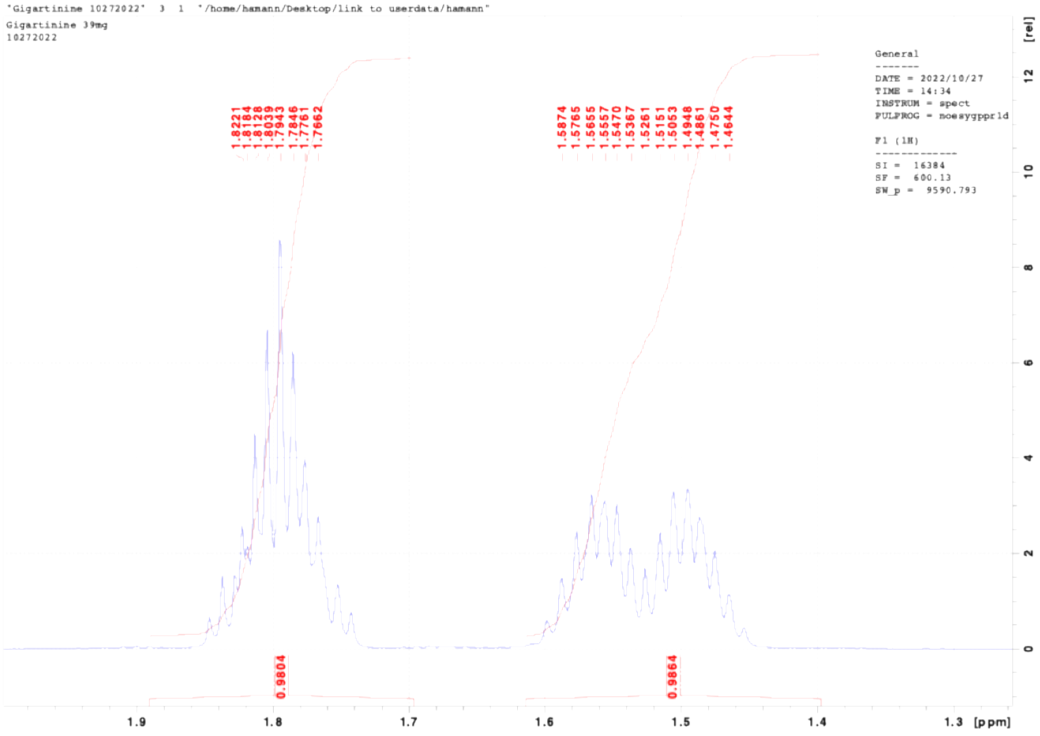

Figure S2: ^{1}H NMR spectra for gigartinine.

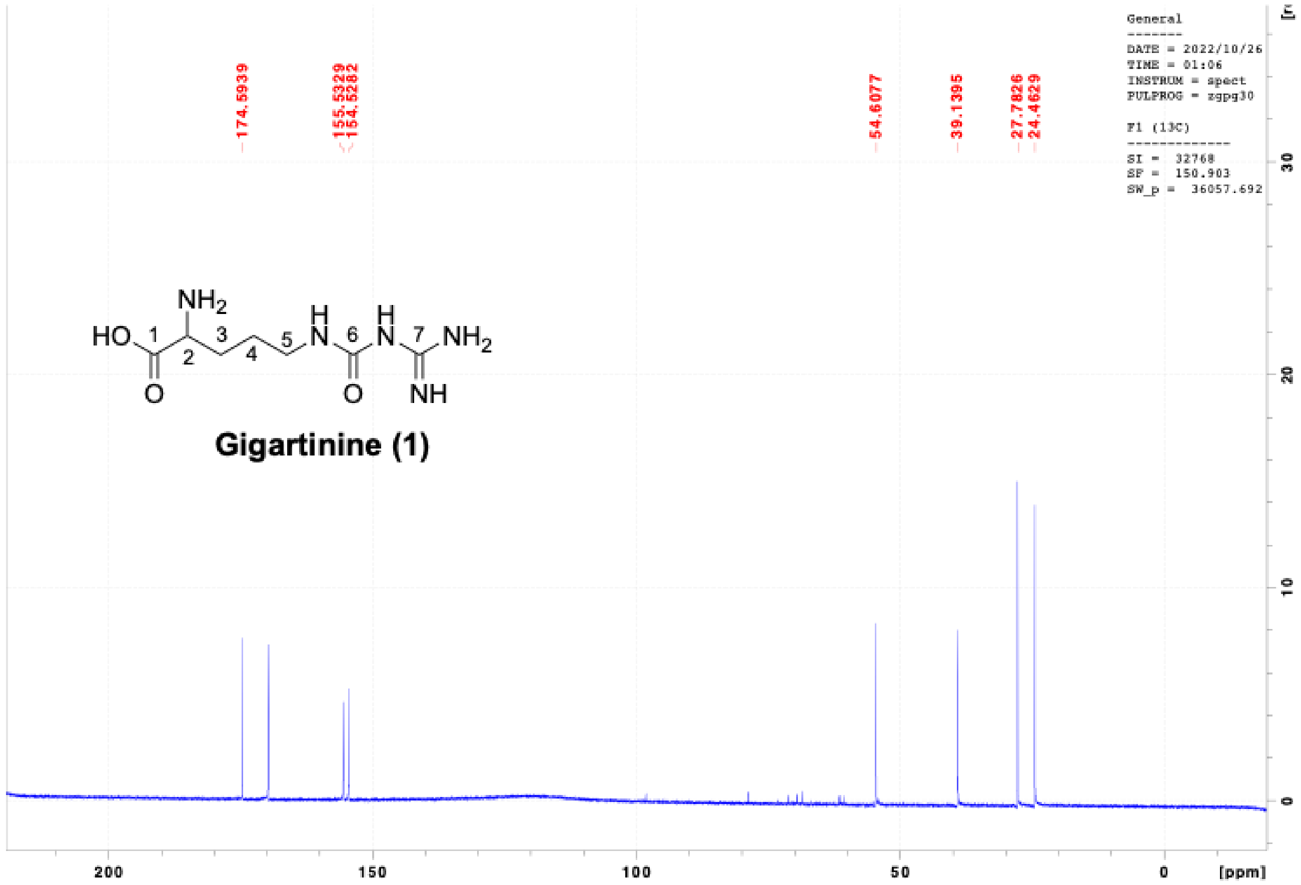

Figure S3: ^{13}C NMR spectra for gigartinine.

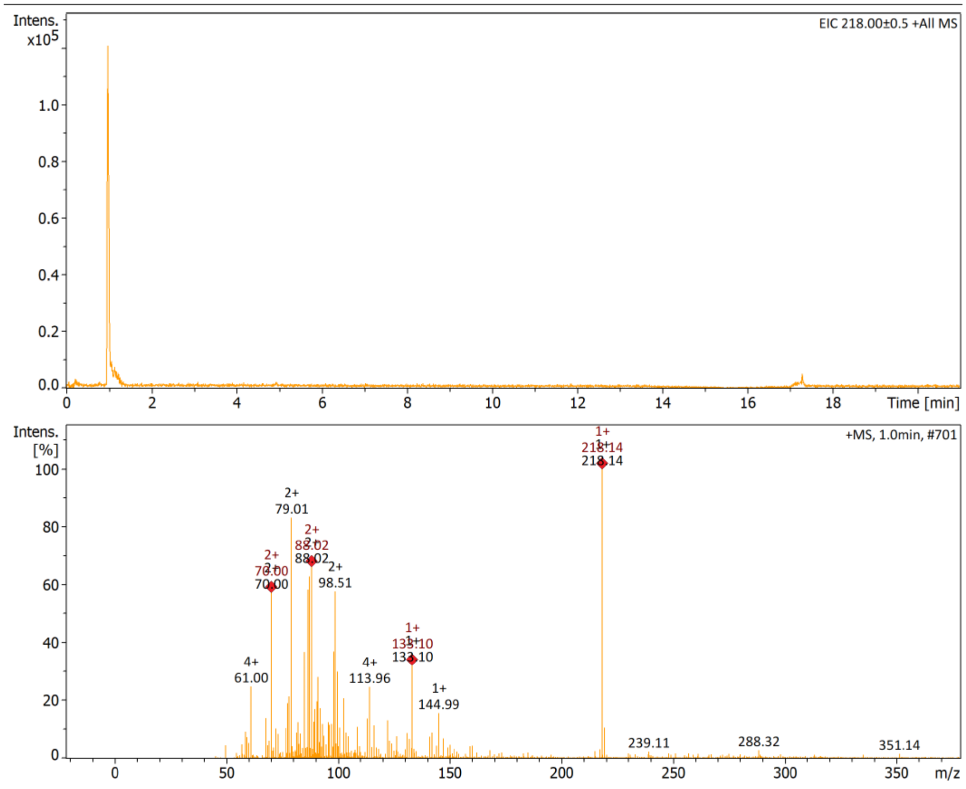

Figure S4: LCMSMS EIC and fragment ion spectrum for gigartinine, m/z 218.14

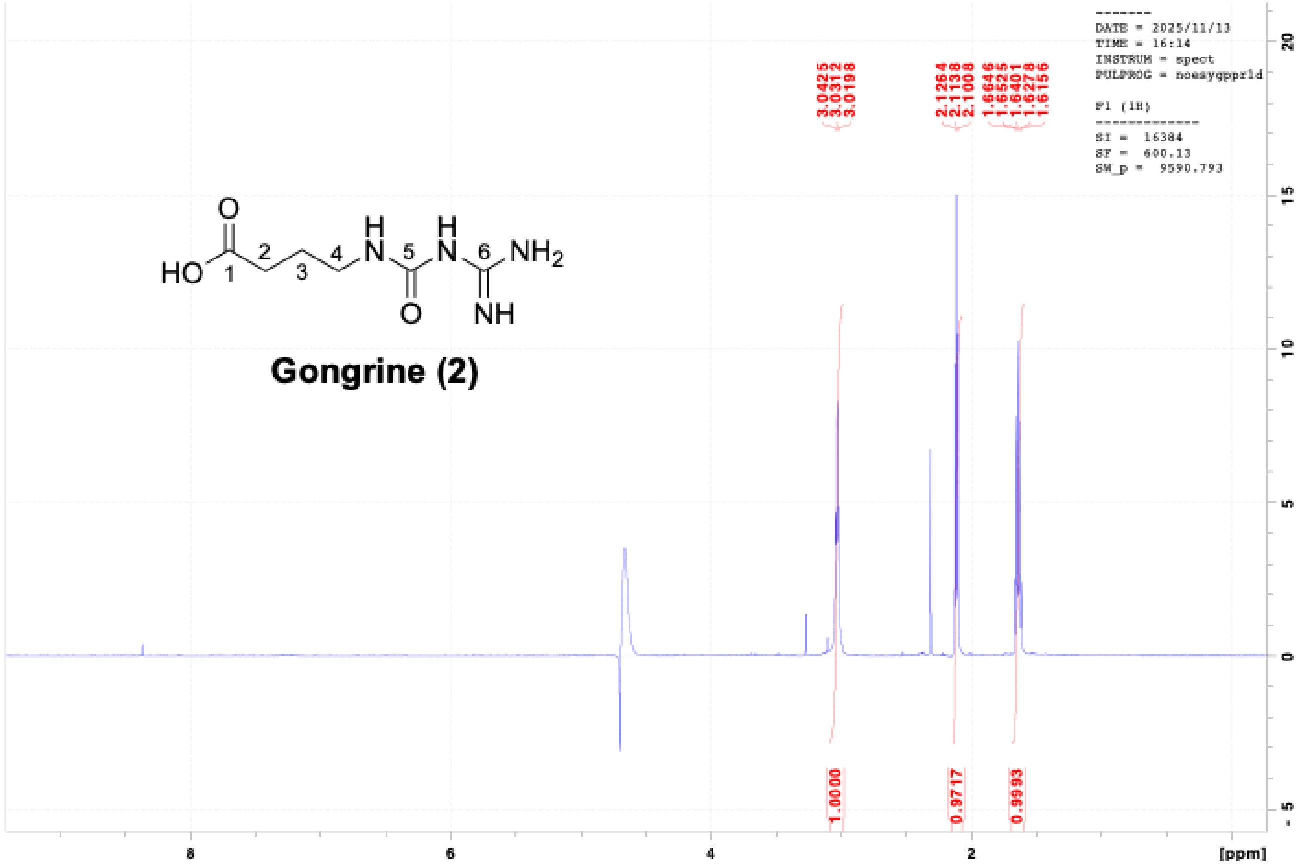
Gongrine (2)
HO
1
2
3
4
N
H
5
O
N
H
6
NH2
NH

DATE = 2025/11/13
TIME = 16:14
INSTRUM = spect
PULPROG = noesygpprld
F1 (1H)
SI = 16384
SF = 600.13
SW_p = 9590.793
3.0425
3.0312
3.0198
2.1264
2.1138
2.1008
1.6646
1.6525
1.6401
1.6278
1.6156
1.0000
0.9717
0.9993
[ppm]

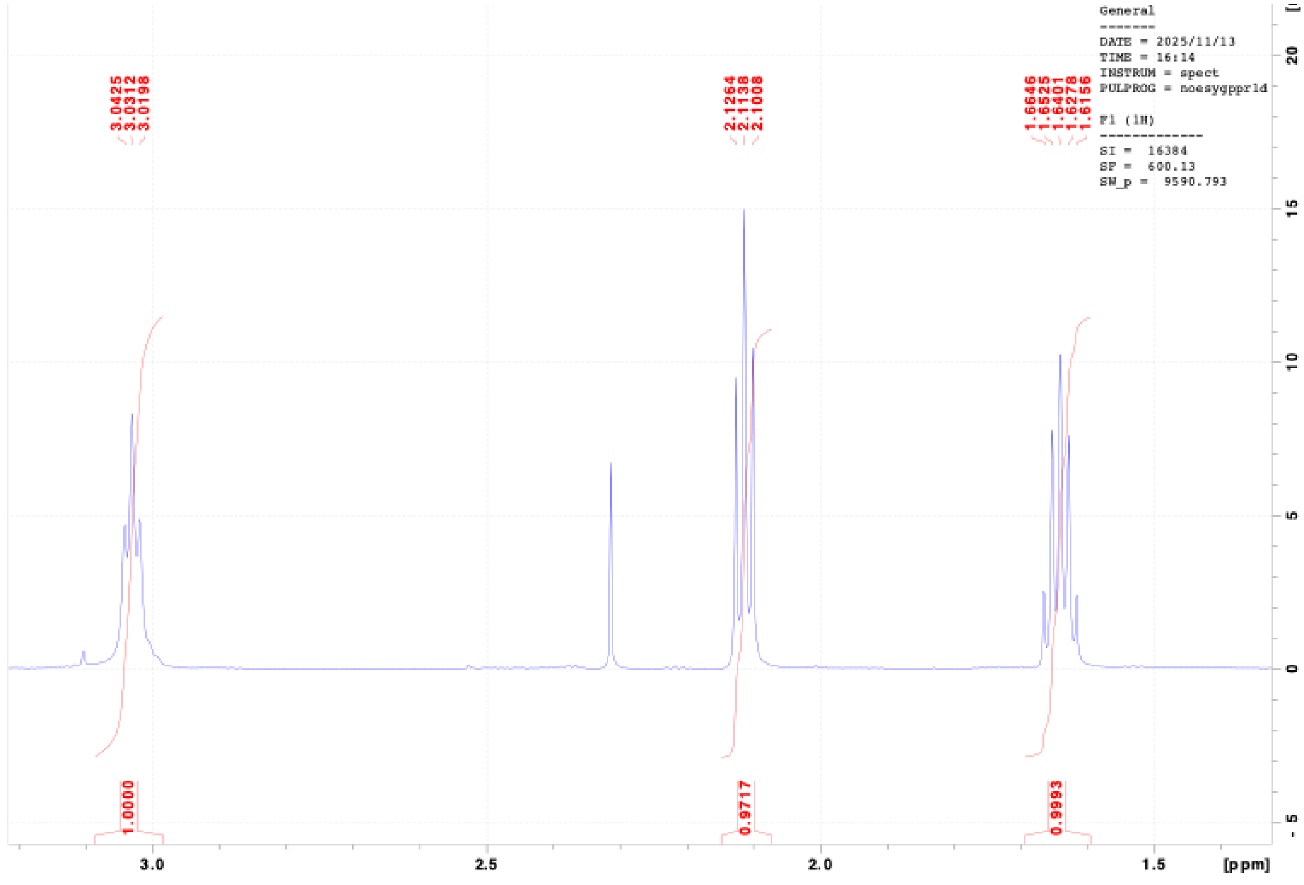
General

DATE = 2025/11/13
TIME = 16:14
INSTRUM = spect
PULPROG = noesygpprld
F1 (1H)
SI = 16384
SF = 600.13
SW_p = 9590.793
3.0425
3.0312
3.0198
2.1264
2.1138
2.1008
1.6646
1.6525
1.6401
1.6278
1.6156
1.0000
0.9717
0.9993
[ppm]

Figure S5: ^{1}H Spectra for gongrine.

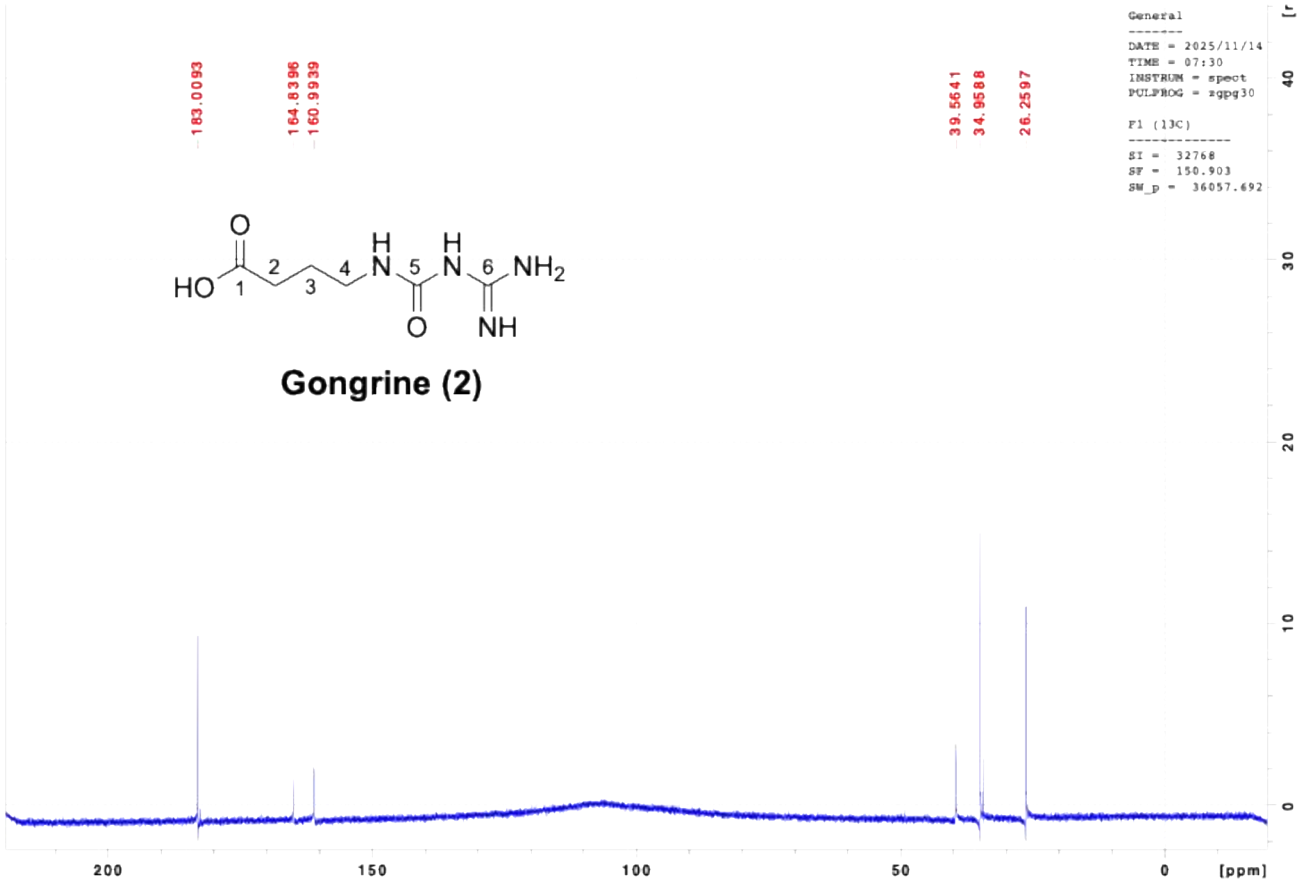

Figure S6: ^{13}C Spectra for gongrine.

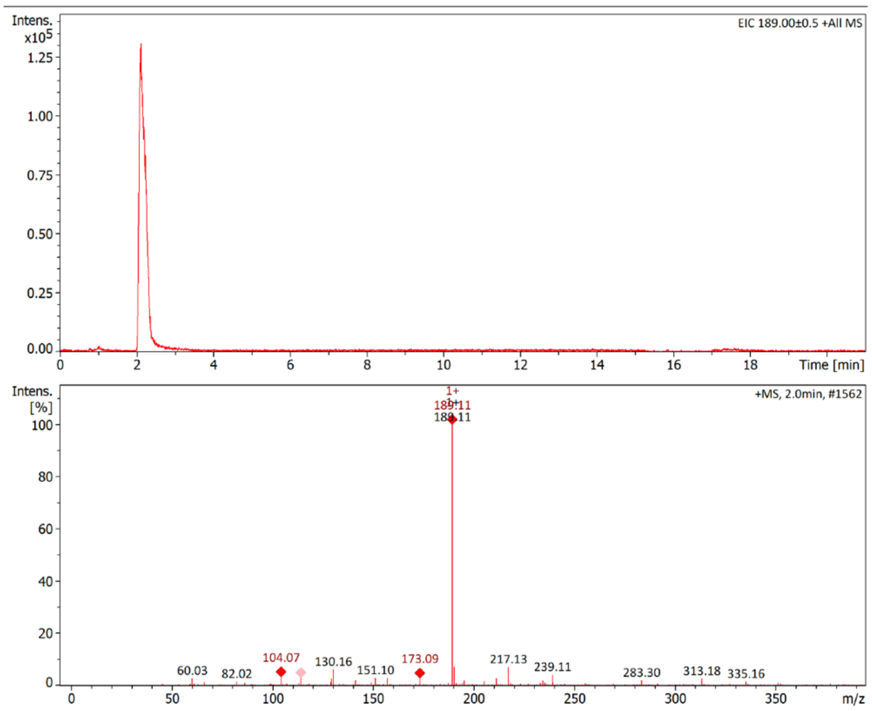

Figure S7: LCMSMS EIC and fragment ion spectrum for gongrine, m/z 189.11

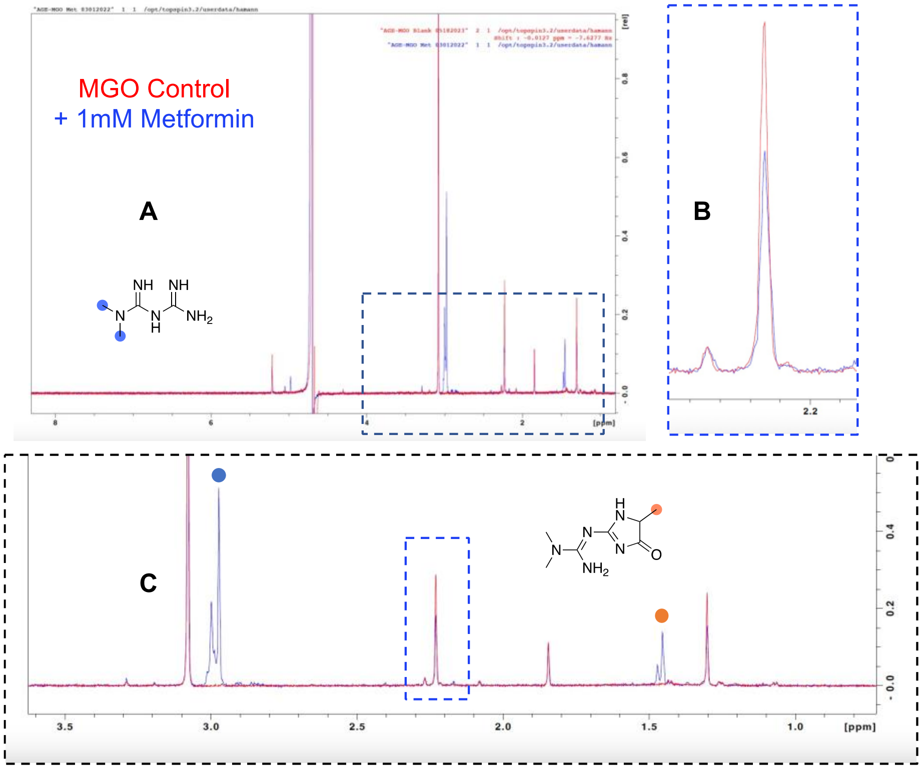

Figure S8: (A) Overlay of MGO control and 1mM Metformin after incubation. (B) MGO signal used for quantification. (C) Indication of key signals (orange dots) and structure of Metformin-MGO reaction product based on previous reports.

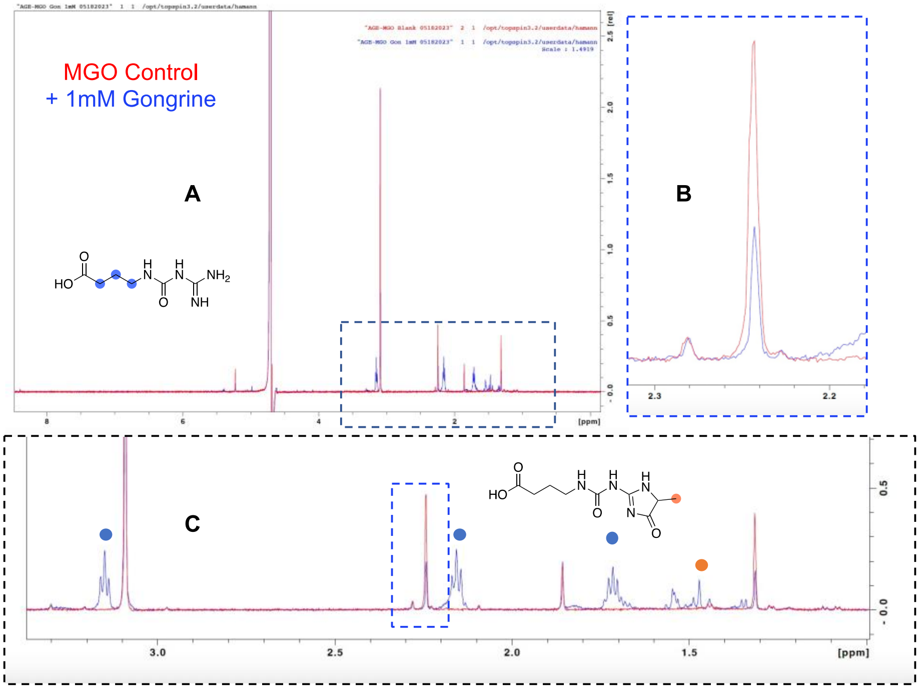

Figure S9: (A) Overlay of MGO control and 1mM Gongrine after incubation. (B) MGO signal used for quantification. (C) Indication of key signals (orange dots) and putative structure of Gongrine-MGO reaction product.

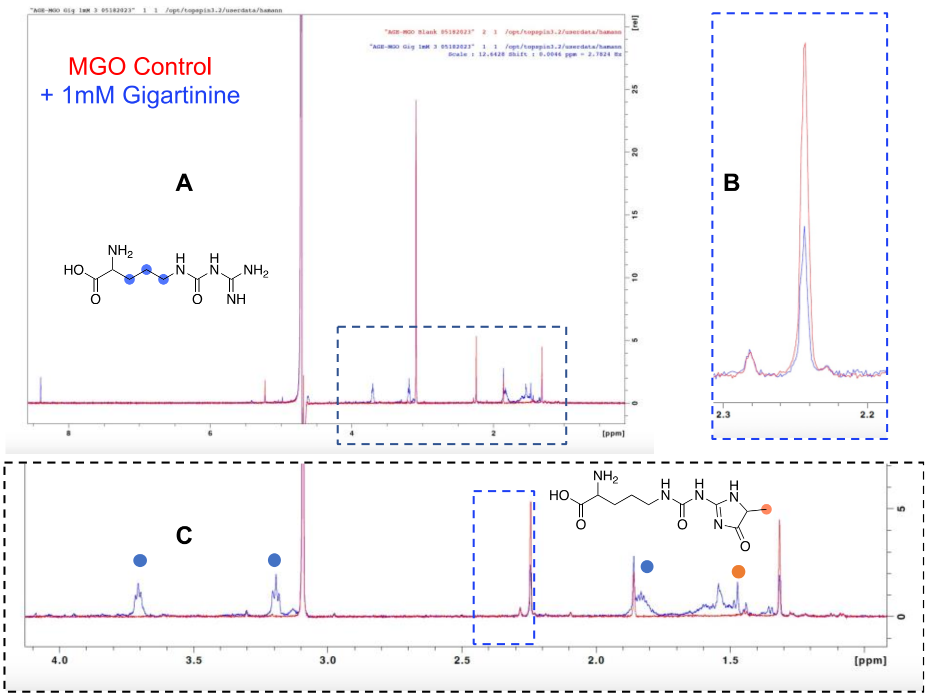

Figure S10: (A) Overlay of MGO control and 1mM Gigartinine after incubation. (B) MGO signal used for quantification. (C) Indication of key signals (orange dots) and putative structure of Gigartinine-MGO reaction product.

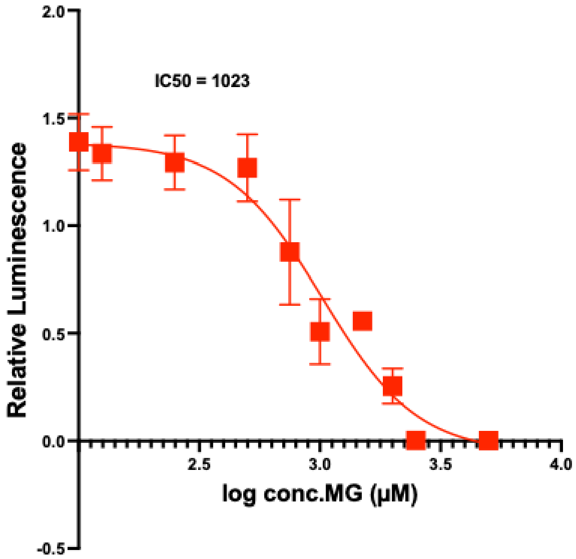

Figure S11: 5000 cells were seeded in a 96 well plate and treated with MGO (0, 100μM to 5mM) for 24 hours. Cell viability was measured using CellTitre-Glo Assay. Each treatment was done in four replicates. X values were transformed using the function X=log(X) and a four-parameter non-linear fit was performed to calculate the IC50.
